# Selective Effects of Thioridazine on Self-Renewal of Basal-Like Breast Cancer Cells

**DOI:** 10.1038/s41598-019-55145-3

**Published:** 2019-12-10

**Authors:** Matthew Tegowski, Cheng Fan, Albert S. Baldwin

**Affiliations:** 1 Curriculum in Genetics and Molecular Biology, The University of North Carolina at Chapel Hill, Chapel Hill, NC 27599 USA; 2 Lineberger Comprehensive Cancer Center, The University of North Carolina at Chapel Hill, Chapel Hill, NC USA

**Keywords:** Breast cancer, Cancer stem cells

## Abstract

Several recent publications demonstrated that DRD2-targeting antipsychotics such as thioridazine induce proliferation arrest and apoptosis in diverse cancer cell types including those derived from brain, lung, colon, and breast. While most studies show that 10–20 µM thioridazine leads to reduced proliferation or increased apoptosis, here we show that lower doses of thioridazine (1–2 µM) target the self-renewal of basal-like breast cancer cells, but not breast cancer cells of other subtypes. We also show that all breast cancer cell lines tested express DRD2 mRNA and protein, regardless of thioridazine sensitivity. Further, DRD2 stimulation with quinpirole, a DRD2 agonist, promotes self-renewal, even in cell lines in which thioridazine does not inhibit self-renewal. This suggests that DRD2 is capable of promoting self-renewal in these cell lines, but that it is not active. Further, we show that dopamine can be detected in human and mouse breast tumor samples. This observation suggests that dopamine receptors may be activated in breast cancers, and is the first time to our knowledge that dopamine has been directly detected in human breast tumors, which could inform future investigation into DRD2 as a therapeutic target for breast cancer.

## Introduction

The five dopamine receptors (DRD1–5) are G-protein-coupled receptors (GPCRs) that mediate responses to the catecholamine dopamine^1,2^. Although primarily studied for roles in neurotransmission, dopamine receptors have peripheral functions in the pituitary^3^, kidney^4^, adrenal glands^1^, as well as in immune cells^5,6^. There are two subtypes of dopamine receptor, the D1-like receptors (DRD1, DRD5) and the D2-like receptors (DRD2, DRD3, DRD4). The D1-like receptors are coupled to G_αs_ proteins and promote cAMP production, while the D2-like receptors are coupled to G_αi/o_ proteins and inhibit cAMP production; thus, these receptors can have opposing effects on cells when activated^1,2^.

Nearly 30 years ago, thioridazine and pimozide, antipsychotic drugs that primarily block dopamine receptor 2 (DRD2), were shown to inhibit the proliferation of breast cancer cell lines^7,8^. More recently, thioridazine was identified in a screen for small molecules that target cancer stem cells (CSCs)^9^. Following that publication, DRD2-targeting antipsychotics thioridazine and haloperidol have been shown to inhibit proliferation, induce apoptosis, or inhibit CSC-like activity in cell types representing brain^10,11^, lung^12^, leukemia^9^, colon^13^, ovarian^14^, and breast cancers^15,16^. Previous work from our group demonstrated that 5–10 µM thioridazine causes cell cycle arrest in 6 triple-negative breast cancer cell lines tested, but that this is independent of DRD2. Additionally, our study showed that thioridazine inhibits self-renewal of certain triple-negative breast cancer cell lines via DRD2 inhibition^16^. Since most studies have not shown which cancer cell types may be more sensitive than others to thioridazine, or other DRD2-targeting antipsychotics, identifying cancer cell types that are most highly sensitive is critical to understanding whether these compounds could be used effectively as cancer therapeutics.

Breast cancer is the most common cause of cancer in women^17^, and has been shown to consist of different molecular subtypes based on gene expression profiling (luminal A, luminal B, HER2^+^, basal-like, and claudin-low)^18,19^. While the molecular subtypes are based on gene expression, they also correlate with clinical characteristics and outcomes. For example, breast cancers are categorized by their expression of certain targetable receptors. Tumors with estrogen receptor expression can be treated with anti-hormonal therapies, and tumors overexpressing the HER2 receptors can be treated with anti-HER2 therapies. However, there are no standard targeted therapies for patients with triple-negative tumors, which lack expression of estrogen receptor, progesterone receptor, and HER2 receptor^20,21^. Further, a vast majority of basal-like and claudin-low tumors are triple-negative^22^, and therefore have no targeted therapy available. We had previously shown that 1–2 μM thioridazine can inhibit the tumorsphere formation of some triple-negative breast cancer cell lines, but not others^16^, and in this study we sought to address whether cells from some breast cancer subtypes are more sensitive than others.

Critical outstanding questions surrounding the potential use of DRD2-targeting antipsychotics in cancer are the identification of tumor types in which these drugs will be most effective and determining how tumor-expressed dopamine receptors are activated. Additionally, to our knowledge, the presence of dopamine has not been demonstrated in human breast tumors. In this study we show that the self-renewal of basal-like breast cancer cell lines is more sensitive to thioridazine than that of other breast cancer cell lines. We show that DRD2 mRNA and protein can be detected in all breast cancer cell lines tested, suggesting DRD2 expression alone cannot be used to predict whether the self-renewal of a cell line will be sensitive to thioridazine. Interestingly, we also show that a DRD2 agonist, quinpirole, promotes self-renewal even in cell lines whose self-renewal is not sensitive to thioridazine. This suggests that DRD2 is activated in the basal-like cell lines, but not in the non-basal-like cell lines. Further, we report the detection of dopamine in human and mouse triple-negative breast tumor samples, showing that tumor-associated dopamine may be functional in human tumors.

## Results

### Thioridazine inhibits the self-renewal of basal-like breast cancer cells

**Figure 1:**
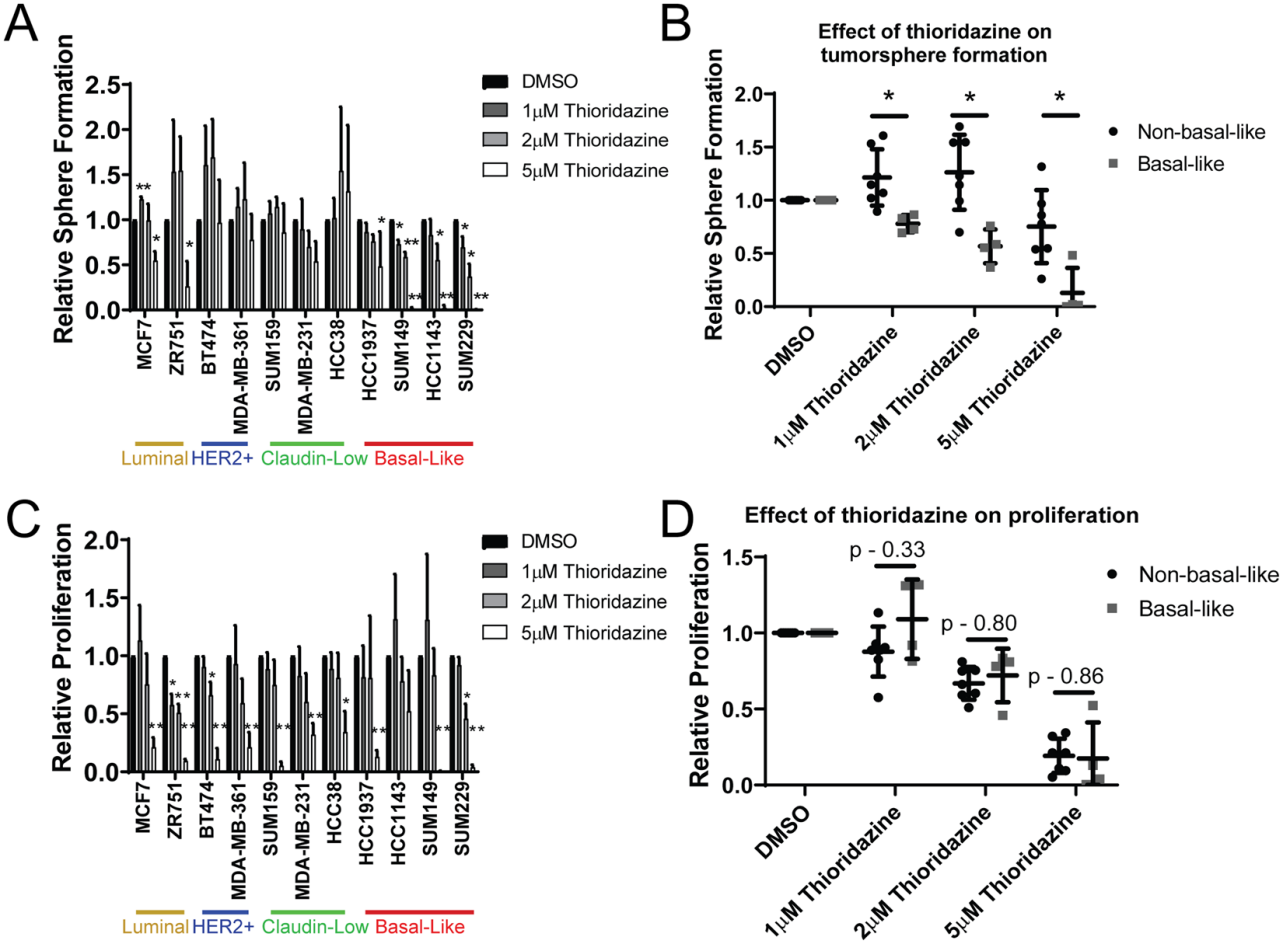
The tumorsphere formation efficiency of basal-like breast cancer cell lines is most sensitive to thioridazine. (**A**) A panel of 11 breast cancer cell lines were cultured in the tumorsphere assay. Cells were treated once with the indicated concentration of thioridazine. 7 days later, the number spheres formed was assessed. Graph is depicted as the fold-change in sphere formation relative to DMSO control for each cell line. The data from SUM159, MDA-MB-231, HCC38, HCC1937, HCC1143, and SUM149 cells was previously published (16) and combined with other cell lines shown. (**B**) The effect of thioridazine on tumorsphere formation of each breast cancer cell line, relative to DMSO, is shown. Non-basal-like cells (black circles) are compared to basal-like cell lines (gray squares). (**C**) A panel of 11 breast cancer cell lines was cultured adherently. Cells were treated once with the indicated concentration of thioridazine. 72 hours later, the number of cells were counted using a hemocytometer. Graph is depicted as the fold-change in cell number relative to DMSO control for each cell line. The data from SUM149 cells was previously published (16) and combined with other cell lines shown. (**D**) The effect of thioridazine on the proliferation of adherent cells of each breast cancer cell line, relative to DMSO, is shown. Non-basal-like cells (black circles) are compared to basal-like cell lines (gray squares). All experiments were performed in biological triplicates. Error bars represent standard deviation. Significance represents a one-sided t-test in (**A**,**C**), testing the difference from DMSO control. Significance in (**B**,**D**) was measured with a two-sided t-test. *p < 0.05, **p < 0.01.

We previously showed that 1 µM thioridazine inhibits self-renewal in some triple-negative breast cancer cell lines through DRD2 inhibition^16^. Specifically, thioridazine inhibited the self-renewal of basal-like cell lines, but not claudin-low cell lines^16^. However, whether the effects of thioridazine on cancer cells are mediated by DRD2 inhibition have been clouded by its extensive polypharmacology^23^. To further investigate the effects of thioridazine on self-renewal, we expanded the experimental panel of breast cancer cell lines to include luminal cell lines (MCF7 and ZR751), HER2+ cell lines (BT474 and MDA-MB-361), as well as another basal-like cell line (SUM229). We cultured these cell lines as tumorspheres, treating them once with DMSO, 1 µM, 2 µM, or 5 µM thioridazine. After 7 days, the number of tumorspheres formed was determined, and the results were compared to those previously published^16^. Interestingly, we observed that low doses of thioridazine (1–2 µM) inhibited tumorsphere formation in 3 out of 4 basal-like cell lines (HCC1143, SUM149, and SUM229), and not in cell lines of other subtypes (Fig. 1A,B). This suggests that thioridazine inhibits self-renewal selectively in basal-like breast cancer cells.

The tumorsphere assay relies on the ability of cells to survive and proliferate. The previously observed cytotoxic effects of thioridazine would reduce tumorsphere formation, even without specifically affecting self-renewal. To test whether proliferation inhibition may affect the tumorsphere assay results, we treated adherently growing cells with DMSO, 1 µM, 2 µM, or 5 µM thioridazine and counted the number of cells after 72 hours. The results for SUM149 cells were previously published^16^. Interestingly, 1 µM thioridazine only reduced proliferation in the luminal ZR751 cells (Fig. 1C), even though tumorsphere formation in this cell line was unaffected. Further, 2 µM does not have a significant effect on proliferation in most cell lines (Fig. 1C,D). In fact, thioridazine significantly decreased the tumorsphere formation of basal-like cell lines compared to the non-basal-like cell lines (Fig. 1B), but thioridazine did not differentially inhibit the proliferation of basal-like relative to non-basal-like cell lines (Fig. 1D) This suggests that 1 or 2 µM thioridazine inhibits the self-renewal of basal-like cell lines, but not proliferation or cell viability. However, 5 µM thioridazine induces a strong decrease in proliferation in all the cell lines (Fig. 1C,D), which we have previously shown to be independent of DRD2 inhibition^16^.

**Figure 2:**
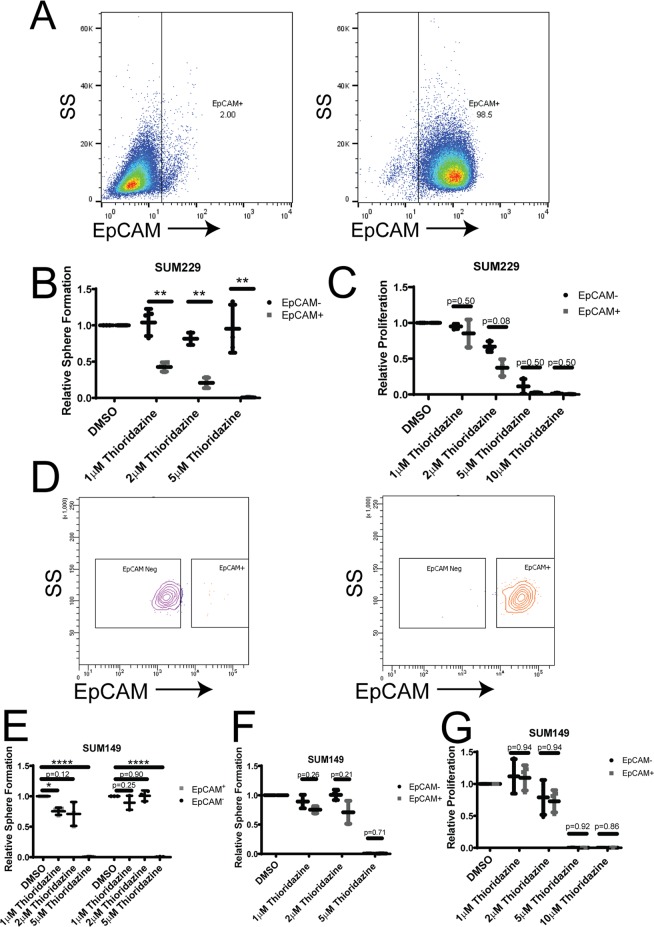
Tumorsphere formation of the basal-like population within cell lines is more sensitive to thioridazine. (**A**) The presence of EpCAM on the cell surface of SUM229-EpCAM^−^ cells (left) and SUM229-EpCAM^+^ cells (right) was assessed using flow cytometry. (**B**) SUM229-EpCAM^−^ and SUM229-EpCAM^+^ cells were cultured in a tumorsphere assay. The cells were treated with the indicated concentrations of thioridazine once upon plating. After 7 days, the number tumorspheres formed was assessed. Graph depicts the fold-change in tumorsphere number relative to DMSO control for each cell line. The effect of thioridazine on the tumorsphere formation of EpCAM^−^ cells (black circles) is compared to the effect on EpCAM^+^ cells (gray squares) (**C**) SUM229-EpCAM^−^ and SUM229-EpCAM^+^ cells were cultured adherently. Cells were treated once with the indicated concentration of thioridazine. 72 hours later, the number of cells were counted using a hemocytometer. The graph depicts the fold-change in cell number relative to DMSO control for EpCAM^−^ cells (black circles) compared to EpCAM^+^ cells (gray squares). (**D**) SUM149-cells were flow-sorted into EpCAM^−^ and EpCAM^+^ populations. The post-sort flow analysis shows the surface expression of EpCAM in SUM149-EpCAM^−^ cells (left) and SUM149-EpCAM^+^ cells (right). (**E**) SUM149-EpCAM^−^ and SUM149-EpCAM^+^ cells were cultured in a tumorsphere assay. The cells were treated with the indicated concentrations of thioridazine once upon plating. After 7 days, the number tumorspheres formed was assessed. Graph depicts the fold-change in tumorsphere number relative to DMSO control for each cell line. (**F**) The graph directly compares the effect of thioridazine on the tumorsphere formation of SUM149-EpCAM^−^ (black circles) and SUM149-EpCAM^+^ cells (gray squares). (**G**) SUM149-EpCAM^−^ (black circles) and SUM149-EpCAM^+^ cells (gray squares) were cultured adherently. Cells were treated once with the indicated concentration of thioridazine. 72 hours later, the number of cells were counted using a hemocytometer. The graph depicts the fold-change in cell number relative to DMSO control, and compares the effect of thioridazine on the proliferation of SUM149-EpCAM^−^ (black circles) and SUM149-EpCAM^+^ cells (gray squares). All experiments were performed in biological triplicate. Error bars represent standard deviation. Significance in (**E**) was measured with a one-sided t-test, testing the difference from DMSO control. Significance in (**B**,**C**,**F**,**G**) was measured with a two-sided t-test. *p < 0.05, **p < 0.01, ***p < 0.001, ****p < 0.0001.

Basal-like breast cancer cell lines have been described to consist of distinct populations of mostly basal-like cells with a subpopulation of claudin-low cells^24^. These populations are derived from the same cell line, but represent different epigenetic and transcriptomic states resembling epithelial basal-like cells (EpCAM^+^) and mesenchymal claudin-low cells (EpCAM^−^)^19,24^. We used SUM229 cells sorted into EpCAM^+^ and EpCAM^−^ populations (Fig. 2A), and tested the tumorsphere formation of each population when treated with DMSO, 1 µM, 2 µM, or 5 µM thioridazine. Strikingly, 1 µM and 2 µM thioridazine strongly decreased tumorsphere formation in the basal-like EpCAM^+^ cells, but not in the claudin-low EpCAM^−^ cells (Fig. 2B). Not only did thioridazine treatment induce a dose-dependent decrease in tumorsphere formation in the EpCAM^+^ cells, but the number of tumorspheres formed was completely unaffected by any tested concentration of thioridazine in the EpCAM^−^ cells (Fig. 2B). Interestingly, 5–10 µM thioridazine strongly inhibited the adherent proliferation of both EpCAM^+^ and EpCAM^−^ cells (Fig. 2C). The observation that 5 µM thioridazine also strongly inhibits proliferation, but has no effect on the number of EpCAM^−^ tumorspheres formed suggests that higher doses of thioridazine inhibit the proliferation of the bulk of tumor cells, but do not inhibit the self-renewal capacity of sphere-forming cells. To test whether this is a broader effect in basal-like breast cancer cells, we sorted another cell line, SUM149, into EpCAM^+^ basal-like cells and EpCAM^−^ claudin-low cells (Fig. 2D). We tested the effect of DMSO, 1 µM, 2 µM, or 5 µM thioridazine on tumorsphere formation in each of these populations. Interestingly, 1 or 2 µM thioridazine treatment led to a statistically significant decrease in tumorsphere formation in EpCAM^+^ cells, but not in EpCAM^−^ cells (Fig. 2E). However, the difference in tumorsphere reduction between the EpCAM^+^ and EpCAM^−^ cells induced by thioridazine was not statistically significant (Fig. 2F). The effect of thioridazine on inhibiting tumorsphere formation of EpCAM^+^ cells was more modest in the SUM149 cells than it was in the EpCAM^+^ SUM229 cells (Fig. 2E,F vs. Fig. 2B). Further, treatment with 5 µM thioridazine nearly eliminated tumorsphere formation in both sorted SUM149 populations (Fig. 2E). Together, with the complete loss of adherent growth of both populations in the presence of 5–10 µM thioridazine (Fig. 2G), this suggests that thioridazine has a stronger effect on overall cell viability of SUM149 cells than SUM229 cells, regardless of whether they resemble basal-like or claudin-low cells. These data show that thioridazine inhibits the self-renewal of basal-like breast cancer cell populations.

### DRD2 is expressed in breast cancer cells

**Figure 3:**
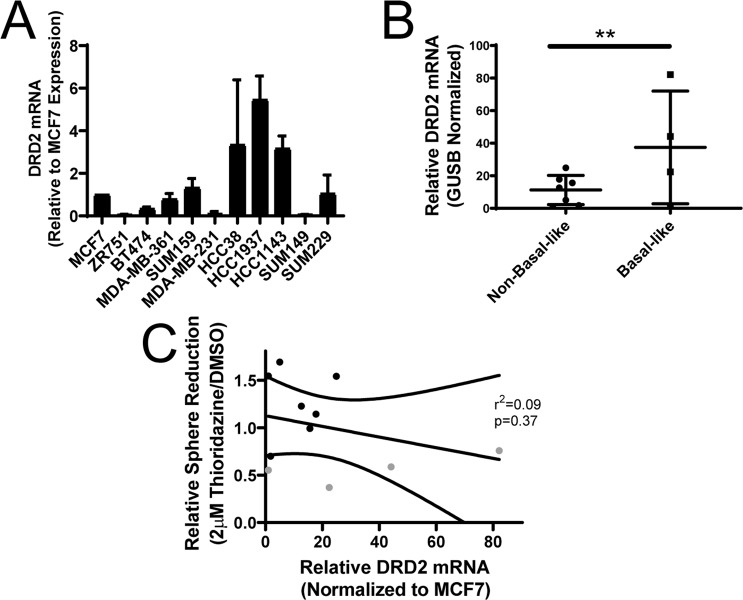
DRD2 mRNA expression in breast cancer cell lines. (**A**) Total RNA was harvested from a panel of 11 breast cancer cell lines, cDNA was made and DRD2 mRNA abundance was measured by qPCR. Graph depicts the average DRD2 expression normalized to GUSB for each cell line, relative to MCF7 cells. (**B**) The average expression of each basal-like and non-basal-like cell line is shown. (**C**) The average DRD2 mRNA expression is shown for each basal-like (gray) and non-basal-like cell line (black) is shown in relation to the effect of 2 µM thioridazine on tumorsphere formation for each cell line. A linear regression was performed to find the line of best fit. Curved lines represent the 95% confidence interval, and the p-value is the likelihood that the slope in non-zero. All experiments were perfomed with biological triplicates. Error bars fpr (**B**) represent standard deviation. Significance for (**B**) was measured using a two-sample t-test. **p < 0.01.

Since we had previously shown that 1–2 µM thioridazine inhibits tumorsphere formation via DRD2 inhibition^16^, we investigated whether the basal-like cells express more DRD2 than cells of other subtypes. Previously, we presented TCGA data that there is higher expression of DRD2 mRNA in human basal-like breast tumors than breast tumors of other subtypes^16^. To probe whether the basal-like cell lines express more DRD2, we harvested total RNA and measured DRD2 mRNA expression by qPCR. Interestingly, in agreement with human tumor data, DRD2 mRNA expression is, on average, elevated in basal-like cell lines (Fig. 3A,B). However, it is important to note that the highly sensitive cell lines SUM229 and SUM149 cells express less DRD2 mRNA than some thioridazine-insensitive cell lines like SUM159 or MCF7 (Fig. 3A). Additionally, HCC1937 cells exhibit the highest relative DRD2 expression (Fig. 3A), even though thioridazine does not affect tumorsphere formation at 1 or 2 µM (Fig. 1A). Further, there is not a significant correlation between relative DRD2 mRNA expression and the effect of thioridazine on tumorsphere formation (Fig. 3C). These data show that DRD2 mRNA expression does not predict whether a cell line is sensitive to DRD2 inhibition.

**Figure 4:**
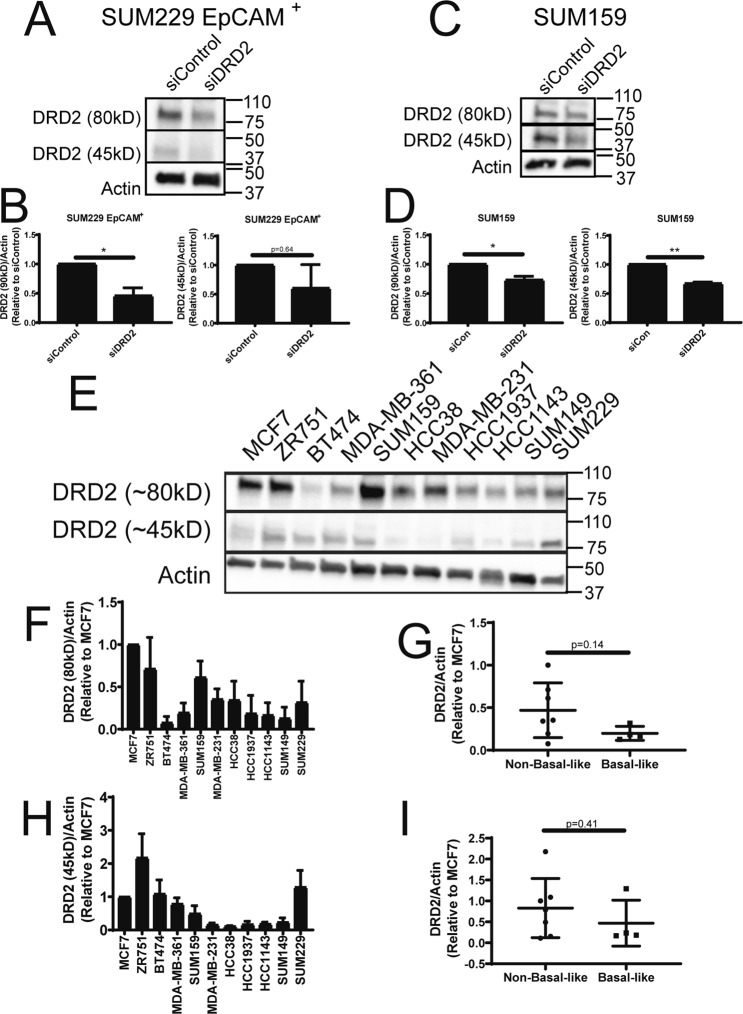
DRD2 protein expression in breast cancer cell lines. (**A**) SUM229-EpCAM^+^ cells were treated with siControl or siDRD2 for 72 hours, lysed, and protein was collected. DRD2 protein was assessed by western blot. (**B**) Actin-normalized quantification of the bands decreased by siDRD2 treatment in (**A**). (**C**) SUM159 cells were treated with siControl or siDRD2 for 72 hours, lysed, and protein was collected. DRD2 protein was assessed by western blot. (**D**) Actin-normalized quantification of the bands decreased by siDRD2 treatment in (**C**). (**E**) The abundance of DRD2 protein in a panel of 11 breast cancer cell lines was assessed by western blot. (**F**,**H**) The relative, actin-normalized abundance of each band was quantified relative to MCF7 cells. (**G**,**I**) The actin-normalized average abundance of each DRD2 band in basal-like and non-basal-like cells was compared. Each experiment was performed in biological triplicate. Error bars represent standard deviation. Significance for (**B**,**D**) was tested using a one-sample t-test relative to siControl. Significance for (**G**,**I**) was tested using a two-sample t-test. *p < 0.05, **p < 0.01.

We investigated whether basal-like cell lines express increased levels DRD2 protein. Few reliable antibodies against DRD2 have been produced. However, a recent study identified one commercially available DRD2 antibody that identifies several proteins that migrate between 70 and 100kD which are not identified in tissues from DRD2 knockout mice^25^. To test whether this antibody detects DRD2-specific bands in breast cancer cell lines we knocked down DRD2 in a thioridazine-sensitive cell line (SUM229-EpCAM^+^) and a thioridazine insensitive cell line that expresses DRD2 mRNA (SUM159), and ran western blots using this anti-DRD2 antibody. Interestingly, we observed a band that migrates around 80kD that is consistently reduced in abundance in the siDRD2 samples in both SUM229-EpCAM^+^ and SUM159 cells (Fig. 4A–D). Additionally, we also observed a band that runs at approximately 45kD, which is close to the molecular weight of DRD2 (52kD). This band is also consistently reduced in the siDRD2 samples in both cell lines (Fig. 4A–D). These molecular weights are also in agreement with previous studies that used photoaffinity labeling to detect DRD2 protein, and likely represent glycosylated and non-glycosylated forms of DRD2^26–28^. We then compared the abundance of these two DRD2 bands across the panel of 11 breast cancer cell lines. Intriguingly, basal-like cell lines do not express elevated DRD2 protein (Fig. 4E–I). Strikingly, there is a trend toward decreased DRD2 protein in basal-like cell lines when compared to cell lines of other subtypes (Fig. 4G,I). Finally, the relative level of DRD2 protein in each cell line was compared to the effect of thioridazine on tumorsphere formation in each cell line. No statistically significant correlation was observed (Fig. S1A,B). Together, these data indicate that DRD2 mRNA and protein can be detected in many breast cancer cell lines. Further, there is no evidence that the level of expression of DRD2 mRNA or protein is predictive of whether thioridazine inhibits self-renewal of a cell line.

### Activated DRD2 can support self-renewal in thioridazine-insensitive cell lines

**Figure 5:**
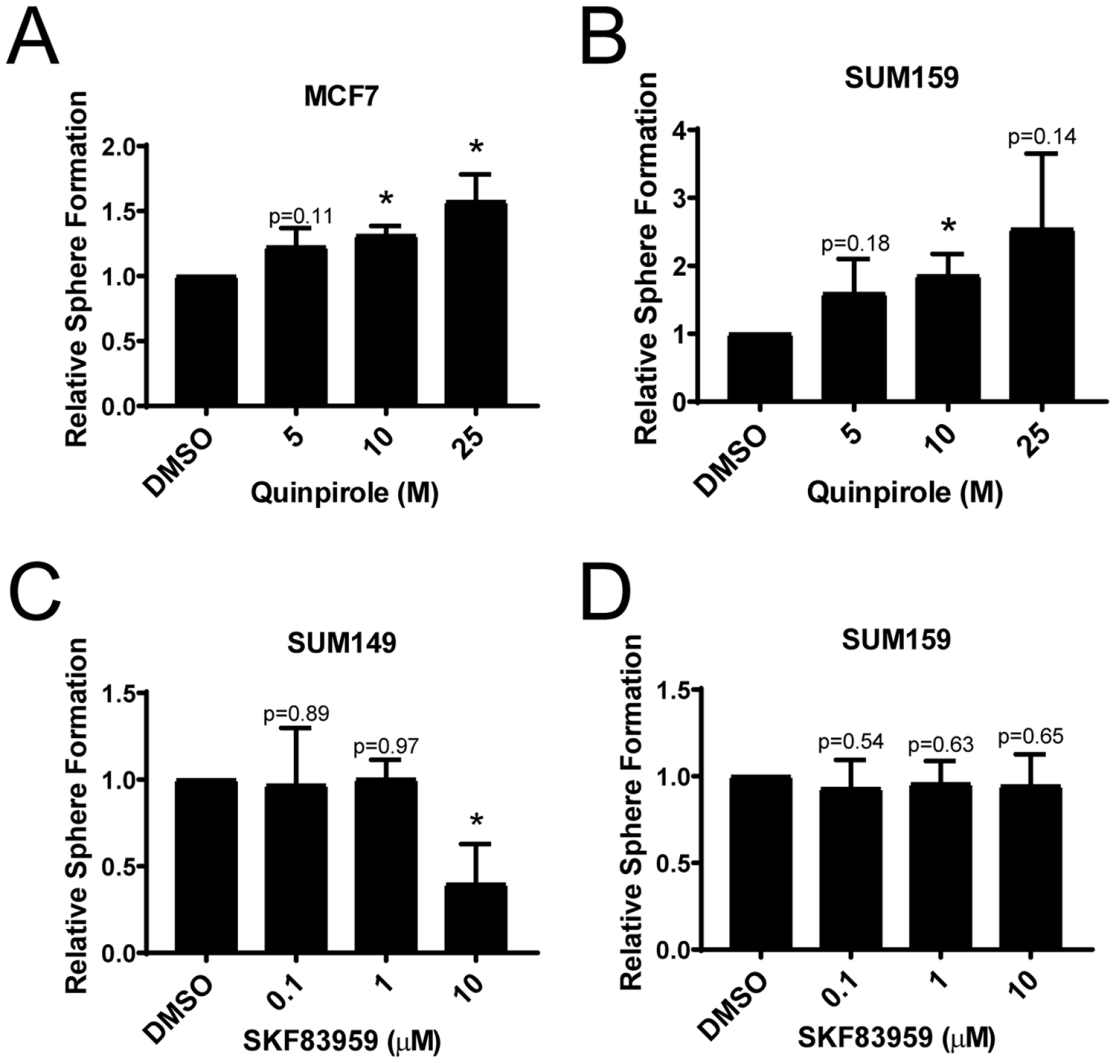
DRD2, but not DRD1, promotes tumorsphere formation in thioridazine-insensitive cell lines. (**A**) MCF7 cells were cultured in the tumorsphere assay. Cells were treated daily with the indicated concentration of quinpirole. After 7 days, the number of tumorspheres formed was assessed. Graph depicts the fold-change in tumorsphere number relative to DMSO. (**B**) SUM159 cells were cultured in the tumorsphere assay. Cells were treated daily with the indicated concentration of quinpirole. After 7 days, the number of tumorspheres formed was assessed. Graph depicts the fold-change in tumorsphere number relative to DMSO. (**C**) SUM149 cells were cultured in the tumorsphere assay. Cells were treated daily with the indicated concentration of SKF83959. After 7 days, the number of tumorspheres formed was assessed. Graph depicts the fold-change in tumorsphere number relative to DMSO. (**D**) SUM159 cells were cultured in the tumorsphere assay. Cells were treated daily with the indicated concentration of SKF83959. After 7 days, the number of tumorspheres formed was assessed. Graph depicts the fold-change in tumorsphere number relative to DMSO. All experiments were performed in biological triplicate. Error bars represent standard deviation. Significance was tested using a one-sample t-test relative to DMSO. *p < 0.05.

DRD2 expression can be detected in all cell lines tested, regardless of whether thioridazine inhibits self-renewal. To examine this further, we tested the effects of DMSO, 5 µM, 10 µM, or 25 µM quinpirole on the tumorsphere formation of MCF7 and SUM159 cells. Quinpirole is an agonist specific for DRD2/3. MCF7 and SUM159 cells were chosen because thioridazine did not affect their tumorsphere formation efficiency (Fig. 1A), yet both express relatively high levels of DRD2 mRNA (Fig. 3A) and protein (Fig. 4E,F,H). Remarkably, quinpirole dose-dependently increased the tumorsphere formation efficiency of both MCF7 and SUM159 cells (Fig. 5A,B). This suggests that DRD2 is not only expressed, but also capable of supporting self-renewal in these cell lines, but that it is not normally sufficiently activated. To test whether this activity is specific to DRD2-like receptors, we also tested the effects of an agonist of DRD1/5, SKF83959, on tumorsphere formation. Unlike quinpirole, SKF84959 did not increase tumorsphere formation in either SUM149 or SUM159 cells (Fig. 5C,D). Interestingly, 10 µM SKF83959 decreased tumorsphere formation in SUM149 cells, suggesting that DRD1 negatively regulates self-renewal in some cell lines. These data provide evidence that DRD2 is expressed and capable of promoting self-renewal in non-basal-like breast cancer cell lines, but that it is not always active in promoting self-renewal.

### Dopamine in human tumor samples

**Figure 6:**
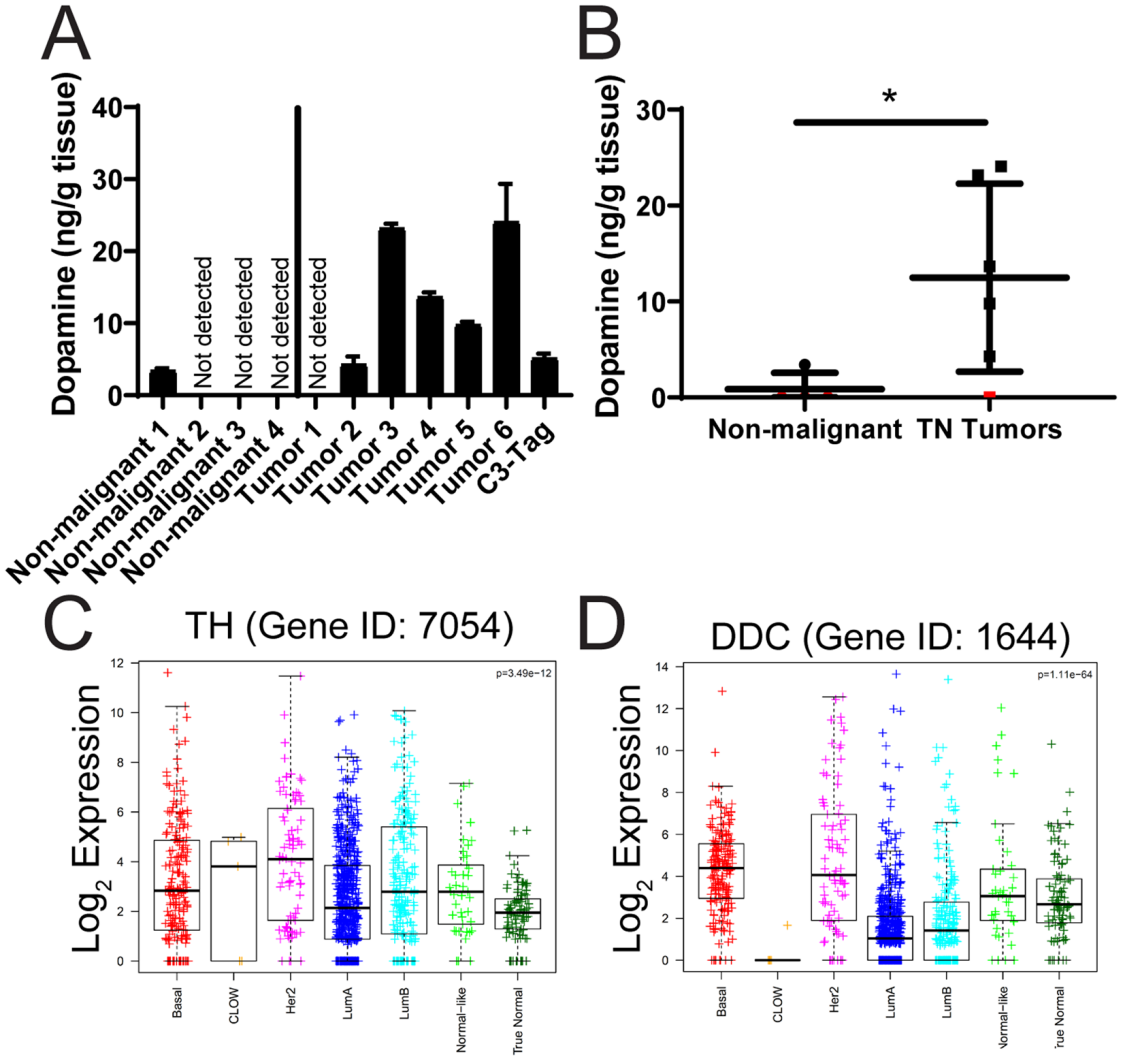
Dopamine abundance in human and mouse tumors. Four non-malignant and six human triple-negative breast tumor samples and a C3-tag mouse tumor were obtained, homogenized in PBS, and the prevalence of dopamine was measured by ELISA. Error bars represent standard deviation of technical triplicates. Each tumor sample represents a separate biological test. (**B**) The average amount of dopamine measured in each human sample is graphed, comparing the non-malignant to the human tumor samples. Samples below detection limit are labeled in red. (**C**,**D**) Log_2_ gene expression of (**C**) TH (Entrez gene ID 7054) and (**D**) DDC (Entrez gene ID 1644) is displayed for each breast cancer subtype by with boxplots. (Basal = basal-like breast cancer; CLOW = claudin-low; Her2 = Her2 amplified; ILC = lobular carcinoma; LumA = Luminal A; LumB = Luminal B). Significance of (**B**) was measured with a two-sided t-test. Bars represent the mean and standard deviation. Significance of (**C**,**D**) was measured using a one-way ANOVA. *p < 0.05.

If DRD2 can promote self-renewal in human tumors, it is important to determine whether dopamine is present in the tumor and/or tumor microenvironment. To test this, we obtained frozen tumor specimens from 6 human triple-negative breast tumors, as well as from a mouse C3-tag tumor, which also has triple-negative characteristics. Interestingly, we were able to detect dopamine in 5 of the 6 human tumors, as well as in the C3-tag murine tumor (Fig. 6A). Interestingly, there was about a 6-fold range in dopamine abundance between the lowest tumor sample (Tumor 2) and the highest (Tumor 6) (Fig. 6A). This indicates that dopamine is present in human breast tumors, and in the mouse C3-tag genetic breast tumor model, and suggests that DRD2 activation could potentially occur downstream of dopamine, supporting self-renewal of breast cancer cells. We also tested the level of dopamine in several non-malignant breast tissue samples. Interestingly, dopamine levels were below the detection limit of our assay in 3 of 4 non-malignant samples, and there was less dopamine detected in the other sample than in most of the tumor samples (Fig. 6A). Further, there was significantly more dopamine in the triple-negative human tumor samples compared with the non-malignant samples (Fig. 6B). Dopamine is synthesized from the amino acid tyrosine via the action of tyrosine hydroxylase (TH) and aromatic amino acid decarboxylase (DDC), and expression of these genes should be detectable if dopamine is being synthesized in the tumors. We examined the expression of TH and DDC across the breast cancer subtypes in the TCGA dataset. Interestingly, both genes are expressed, with wide variability, in breast cancers of all subtypes (Fig. 6C,D). Intriguingly, DDC, the final enzyme that leads to dopamine production, is expressed most in basal-like and HER2+ tumors (Fig. 6D). Overall these data show that dopamine can be produced in breast tumors and/or the tumor microenvironment.

## Discussion

We previously showed that 1–2 µM thioridazine requires DRD2 to inhibit self-renewal in some breast cancer cell lines^16^. Here, we show that basal-like breast cancer cells are selectively sensitive to thioridazine (Fig. 1). Importantly, by sorting EpCAM^+^ and EpCAM^−^ cells, we demonstrated that the self-renewal of the basal-like population of cells from within the same cell line is inhibited by thioridazine, while the self-renewal of the claudin-low cells is not affected (Fig. 2). Further, we demonstrated that DRD2 mRNA and protein can be detected in all cell lines, regardless of molecular subtype and sensitivity to thioridazine (Figs. 3 and 4). Even though basal-like cell lines on average express more DRD2 mRNA, SUM149 and SUM229 cells express lower levels of DRD2 mRNA than SUM159 or MCF7 cells (Fig. 3), in which DRD2 inhibition does not affect tumorsphere formation. Additionally, basal-like cell lines tended to have less DRD2 protein than other cell types (Fig. 4). These data indicate that, while basal-like cells may tend to show increased expression of DRD2 mRNA, DRD2 mRNA or protein abundance alone may not necessarily indicate whether DRD2 actively promotes the self-renewal of a cancer cell line.

We also attempted to uncover why some cell lines are sensitive to thioridazine, while others are not. Thioridazine binds DRD2, and the other D2-like receptors DRD3 and DRD4 with high affinity, it has also been shown to bind other GPCRs, including the D1-like receptors DRD1 and DRD5^23^. Although we have previously shown that 1–2 µM thioridazine requires DRD2 to inhibit self-renewal in basal-like breast cancer cell lines, thioridazine may block self-renewal through the inhibition of other receptors. To approach this question, we tested the effect of highly specific agonists of DRD1/5 (SKF84959) and DRD2/3 (quinpirole) on self-renewal. We observed that specific activation of DRD2 with quinpirole was able to increase tumorsphere sphere formation in SUM159 and MCF7 cells (Fig. 5A,B). Importantly, this occurs even though thioridazine does not inhibit tumorsphere formation in these cell lines. This, together with the gene expression data, suggests that DRD2 is expressed in these cells, and capable of signaling to promote self-renewal, but that it is not being activated. When activated with quinpirole, DRD2 can then promote tumorsphere formation. Interestingly, this seems to be specific to DRD2, as a DRD1-specific agonist, SKF83959, did not increase tumorsphere formation (Fig. 5C,D). It is intriguing to speculate that the basal-like cell lines are releasing dopamine, leading to DRD2 activation through an autocrine mechanism, while the cell lines from other breast cancer subtypes are not. However, we were unable to detect dopamine release to the supernatants of the cell lines. Further analysis using HPLC should be done to test this possibility. Relative to the differential effects seen between the EpCAM^+^ and EpCAM^−^ populations, if the basal-like EpCAM^+^ cells are the cells primarily releasing dopamine then this could explain why thioridazine can inhibit the self-renewal of the parental and EpCAM^+^ populations, but not the EpCAM^−^ cells when cultured in isolation from the basal-like EpCAM^+^ cells. Clearly, more work is needed to confirm the release of dopamine from the basal-like cells.

Since DRD2 shoule be activated in order to support the self-renewal of breast cancer cells, and DRD2 activity is stimulated primarily by dopamine, we sought to determine whether dopamine itself could be detected in cell lines and human breast cancer tissue. Specifically, we were interested in testing whether basal-like cell lines release more dopamine than the cell lines of other subtypes. Therefore, we collected supernatants from several basal-like and non-basal-like cell lines and measured released dopamine with a commercial ELISA assay. While there was a dopamine signal detected in the cell media, it was below the lowest standard and the level of confident detection of the ELISA (data not shown).

There is evidence that WNT5A is able to activate DRD2 independent of dopamine^29^. Further, there is evidence of cooperativity between DRD2 and epidermal growth factor (EGFR)^10,30^. However, dopamine is the primary activator of dopamine receptors *in vivo*. We reasoned that if DRD2 is driving the self-renewal of cancer cells *in vivo*, dopamine may be present in human tumors. So, we obtained samples of human triple-negative breast tumors and non-malignant human breast tissue, as well as a triple-negative mouse C3-tag tumor. Dopamine was detected in five of the human tumors plus the mouse tumor, using ELISA, while dopamine was only detected in one of four non-malignant samples (Fig. 6A,B). If dopamine is present in human tumors, then DRD2-targeting agents could potentially have therapeutic benefit in the clinic.

Although we were able to identify dopamine in human tumor samples, the source of the dopamine is unclear. Even though expression of the dopamine synthesis genes TH and DDC can be identified in human tumors in the TCGA dataset (Fig. 6C,D), that information comes from bulk RNA-seq analysis of human tumor sections. Thus, dopamine synthesis could be generated in tumor cells and/or stromal cells of the tumor microenvironment. The infiltration of immune cells into the tumor microenvironment is a highly studied topic, and infiltration of immune suppressing cells, especially Th2-polarized T helper cells, T regulatory cells and M2-polarized macrophages, is associated with tumor progression and cancer stem cell maintenance^31,32^. Additionally, immune cells such as T cells, B cell and macrophages have been shown to produce dopamine, as well as express dopamine receptors^6,33,34^. Since dopamine has been observed in these immune cells that have important functions within the tumor microenvironment, they could be a source of dopamine *in vivo*, helping to support the self-renewal of tumor cells that express DRD2. Additionally, recent work in mouse models of prostate cancer have shown that tumor innervation, the growth of neurons into the tumor area, promotes angiogenesis and metastasis^35,36^. Interestingly, TH^+^ nerve fibers have been observed in mouse orthotopic and spontaneous genetic models of breast cancer^37^, and the selective depletion of dopaminergic and adrenergic neurons by 6-hydroxydopamine (6-OHDA) treatment reduces the growth of 4T1 breast tumors^37^. Surprisingly, this study also showed that 6-OHDA treatment reduced tumor-associated macrophages, as well as tumor IL-6 production^37^. Our previous study showed that DRD2 activity in cancer cell lines promotes STAT3 activation and IL-6 transcription in order to promote self-renewal^16^. Together, these data suggest that tumor cells, tumor-associated immune cells, and/or tumor-associated neurons could all be potential sources of dopamine in human breast tumors, and this may support the self-renewal of a subset of tumor cells. Further work is required to elucidate the origin of tumor-associated dopamine and to determine whether therapeutic benefits may come from inhibition of dopamine receptors or catecholamine synthesis in tumors.

## Methods

### Cell culture and reagents

All cell lines were obtained from ATCC except SUM149 and SUM159 cells were obtained from the lab of Dr. Charles Perou, and SUM229 parental and EpCAM-sorted cells were obtained from the lab of Dr. Gary Johnson. SUM149, SUM159, SUM229, and EpCAM-sorted SUM149 and SUM229 cells were cultured in HuMEC medium (Gibco, Waltham, MA, USA) with supplements added and 5% FBS. MDA-MB-231 cells were cultured in DMEM (Gibco) with 10% FBS. All other cell lines were cultured in RPMI-1640 (Gibco) with 10% FBS. Penicillin/Streptomycin (Gibco) was added to all culture media. Cells were occasionally tested for mycoplasma with the MycoAlert Mycoplasma Detection Kit (Lonza, Basel, Switzerland). DRD2 antibody was obtained from Millipore (AB5084P) and used at 1:1000. Actin antibody was obtained from Cell Signaling Technologies. Thioridazine and quinpirole were obtained from Sigma-Aldrich, and SKF83959 was obtained from Cayman Chemical.

### Mammosphere assay

The mammosphere assay was performed using Mammocult (Stem Cell Technologies, Vancouver, Canada) as previously described^16^. Briefly, 20,000 cells were cultured in ultra-low adherence 6 well plates in triplicate. The culture was allowed to grow for 7 days before the number of spheres greater than 60 µM in diameter were assessed. Thioridazine treatment was applied once immediately upon plating. Quinpirole and SKF83959 was applied daily.

### Adherent proliferation assay

4,000 cells were plated per well in a 24 well plate in triplicate. Cells were treated once, immediately after plating with the stated concentration of thioridazine. 72 hours later, the cells were trypsinized and counted using a hemacytometer.

### EpCAM flow cytometry

To determine the percentage of EpCAM^+^ and EpCAM^−^ cells, cells were dissociated with TrypLE (Gibco), and 10^6^ cells were added to 100 µL of flow buffer, 1x PBS (Gibco) with 1% BSA. 5 µL of anti-EpCAM-FITC antibody (Stem Cell Technologies) was added and incubated on ice for 1 hour. Cells were washed 3 times with flow buffer, fixed with 10% formalin, passed through a 40 µM filter, and run on a BD Cyan Flow Cytometer. The data were analyzed using FloJo software.

### EpCAM sorting

SUM229-EpCAM^+^ and SUM229-EpCAM^−^ cells were a generous gift from the laboratory of Dr. Gary Johnson. 10^7^ SUM149 cells were dissociated using TrypLE. Cells were treated with DNaseI (Promega, Madison, WI, USA) for 15 minutes in HBSS at room temperature. Subsequently, SUM149 cells were resuspended in 1 mL of flow buffer and 50 µL of anti-EpCAM-FITC antibody (Stem Cell Technologies) and incubated on ice for 1 hour. Cells were washed 3 times with flow buffer with 1 mM EDTA added, passed through a 40 µM filter, and run on a BD FACSAriaII by the UNC Flow Cytometry Core Facility. Post-sort analysis on sorted fractions was performed to determine purity of fractions.

### qPCR

Total RNA was harvested using Trizol according to manufacturer’s protocol (Invitrogen). cDNA was subsequently made using iScript cDNA synthesis kit (BioRad, Hercules, CA, USA). cDNA was used in qPCR reactions run using iTaq universal probes supermix buffer (BioRad) on a ViiA 7 Real-Time PCR System (Thermo-Fisher, Waltham, MA, USA). DRD2 probe was obtained through BioRad, and GUSB probe was obtained through Applied Biosystems (Grand Island, NY, USA).

### Western blots

Western blots were performed as previously described (16). Briefly, cells were lysed for 15 min. on ice in RIPA lysis buffer with cOmplete protease inhibitors (Promega) and phosphatase inhibitor cocktail 3 (Sigma). Insoluble material was pelleted out, and 1 µg/µL samples were made and run on PROTEAN TGX SDS-PAGE gels (Bio-rad) and transferred onto PVDF membranes, and blocked with 5% nonfat dry milk for 1 hour at room temperature. Primary antibodies were added overnight at 4 degrees celcius, then washed with TBST. HRP-conjugated secondary antibodies (Promega) were added for 1 hour at room temperature at 1:10,000 dilution. Membranes were then washed with TBST, and visualized using Clarity ECL (BioRad) on a ChemiDoc system (BioRad).

### Dopamine ELISA

Dopamine ELISA kit was obtained from BioVision (Milpitas, CA, USA), and used according to manufacturer’s protocol. Briefly, frozen human tumor samples and a C3-tag tumor section were weighed and then homogenized in PBS at a concentration of 9 mL of PBS per 1 g tumor tissue. Samples were centrifuged and 50 µL of each sample was used per well in the provided 96 well plate. Subsequent steps were performed according to manufacturer’s protocol. Displayed results are the average of technical replicates from the same sample, and error bars indicate standard deviation.

### Human tumor samples

Human triple-negative tumor samples and normal tissue were generously provided by the laboratory of Dr. Qing Zhang without any identifying information (IRB#15-0041).

### TCGA analysis

The expression of TH (Entrez gene ID: 7054) and DCC (Entrez gene ID: 1644) across breast cancer subtypes was analyzed in the breast invasive carcinoma samples in the TCGA dataset. The subtype calls are from the PAM50 50-gene subtype analysis as described previously^38^. A one-way ANOVA was performed to compare the gene expression values in multiple groups, which were then displayed by boxplot.

## Supplementary information

Figure S1

## Data Availability

No datasets were generated during the course of this study.
