# Supplementary material for: Selective Effects of Thioridazine on Self-Renewal of Basal-Like Breast Cancer Cells
Source: Sci Rep. 2019 Dec 10;9:18695. doi: 10.1038/s41598-019-55145-3 (PMC6904738; doi:10.1038/s41598-019-55145-3)
Supplement: Supplementary file 1 — Figure S1 [file 41598_2019_55145_MOESM1_ESM.pdf]

Selective Effects of Thioridazine on Self-Renewal of Basal-Like Breast Cancer Cells

Matthew Tegowski[1,2], Cheng Fan[2], and Albert S. Baldwin[1,2*]

[1]Curriculum in Genetics and Molecular Biology, The University of North Carolina at Chapel Hill, Chapel Hill, NC, 27599, USA;
[2]Lineberger Comprehensive Cancer Center, The University of North Carolina at Chapel Hill, Chapel Hill, NC, USA.
*To whom correspondence should be addressed: abaldwin@med.unc.edu

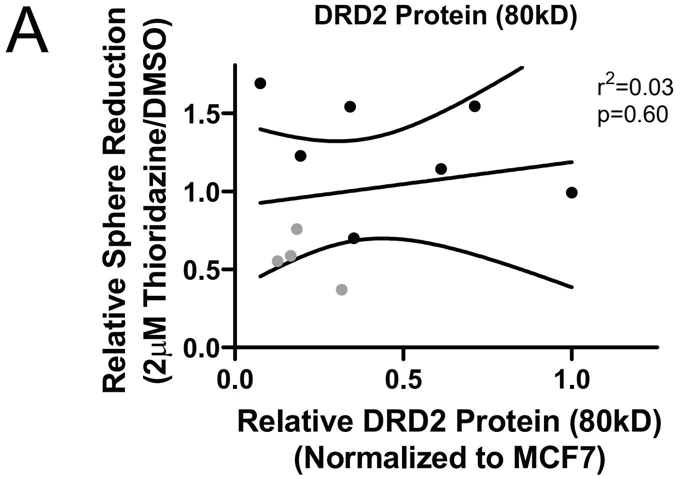

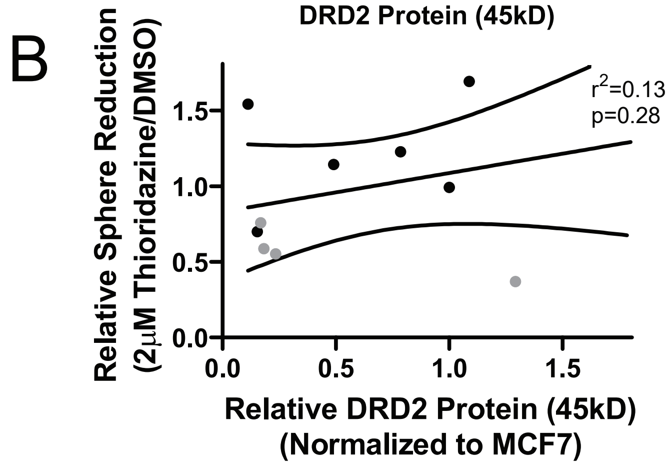

Supporting Figure 1 – DRD2 protein level does not predict effects of thioridazine on tumorsphere formation. (A) The average expression of the ~80KD DRD2 band is shown, relative to MCF7 expression, for each basal-like (gray) and non-basal-like cell line (black) is shown in relation to the effect of 2μM thioridazine on tumorsphere formation for each cell line. A linear regression was performed to find the line of best fit. (B) The average expression of the ~45kD DRD2 band is shown, relative to MCF7 expression, for each basal-like (gray) and non-basal-like cell line (black) is shown in relation to the effect of 2μM thioridazine on tumorsphere formation for each cell line. A linear regression was performed to find the line of best fit. Curved lines represent the 95% confidence interval, and the p-value is the likelihood that the slope in non-zero.
